# The Application of a Bone Marrow Mesenchymal Stem Cell Membrane in the Vascularization of a Decellularized Tracheal Scaffold

**DOI:** 10.1155/2021/6624265

**Published:** 2021-03-05

**Authors:** Zhi-Ye Yao, Bo-Wen Feng, Cai-Sheng Liu, Yu-Mei Liu, Hai-Yu Zhou, Xiao-Hui Zhang, Min-Qiao Jian, Jian-Ling Mo, Yi-Jing Liang, Liang Chen, Xiao-Qing Liu, Yan-Ling Chen, Zhan-Song Zhang, Shao-Ru He

**Affiliations:** ^1^Department of Neonatology, Guangdong Provincial People's Hospital, Guangdong Academy of Medical Sciences, Guangzhou 510030, China; ^2^The Second School of Clinical Medicine, Southern Medical University, Guangzhou 510515, China; ^3^Medical College, South China University of Technology, Guangzhou 510641, China; ^4^Department of Thoracic Surgery, Guangdong Provincial People's Hospital, Guangdong Academy of Medical Sciences, Guangzhou 510030, China; ^5^Department of Cardiology, Guangdong Provincial Cardiovascular Institute, Guangzhou 510030, China; ^6^Department of Epidemiology, Guangdong Provincial People's Hospital, Guangzhou 510030, China

## Abstract

Airway stenosis is a common problem in the neonatal intensive care unit (NICU) and pediatric intensive care unit (PICU). A tissue-engineered trachea is a new therapeutic method and a research hotspot. Successful vascularization is the key to the application of a tissue-engineered trachea. However, successful vascularization studies lack a complete description. In this study, it was assumed that rabbit bone marrow mesenchymal stem cells were obtained and induced by ascorbic acid to detect the tissue structure, ultrastructure, and gene expression of the extracellular matrix. A vascular endothelial cell culture medium was added *in vitro* to induce the vascularization of the stem cell sheet (SCS), and the immunohistochemistry and gene expression of vascular endothelial cell markers were detected. At the same time, vascular growth-related factors were added and detected during SCS construction. After the SCS and decellularized tracheal (DT) were constructed, a tetrandrine allograft was performed to observe its vascularization potential. We established the architecture and identified rabbit bone marrow mesenchymal stem cell membranes by 14 days of ascorbic acid, studied the role of a vascularized membrane in inducing bone marrow mesenchymal stem cells by *in vitro* ascorbic acid, and assessed the role of combining the stem cell membranes and noncellular tracheal scaffolds *in vivo*. Fourteen experiments confirmed that cell membranes promote angiogenesis at gene level. The results of 21-day *in vitro* experiments showed that the composite tissue-engineered trachea had strong angiogenesis. *In vivo* experiments show that a composite tissue-engineered trachea has strong potential for angiogenesis. It promotes the understanding of diseases of airway stenosis and tissue-engineered tracheal regeneration in newborns and small infants.

## 1. Introduction

Tracheal stenosis (TS) refers to the stenosis narrowing of the tracheal lumen caused by various factors, which are divided into congenital and acquired [1, 2]. Often caused by the fusion of the tracheal cartilage ring and a tracheal membrane defect, severe congenital TS is one of an important cause of death in infants, especially neonates. Long-term airway stenosis in infants [2] would bring serious respiratory distress, repeated hospitalization, endotracheal intubation, ventilator support, and respiratory tract infection. At present, the main treatment of congenital TS is by surgery [3]. With the development of surgical technology, sliding tracheoplasty is an effective treatment for patients with long-term severe tracheal stenosis. However, the long-term prognosis is not ideal, with an overall mortality rate of 16%–36% and a reintervention rate as high as 44% [4, 5]. In addition, there are still difficulties in surgery and some problems in operation difficulty, respiratory strategy, and postoperative nursing [5].

**Figure 1:**
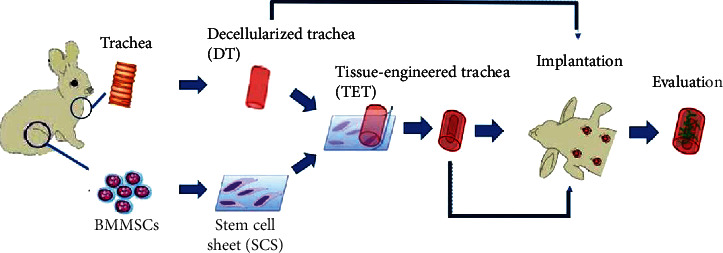
Flow chart of all the experiments.

The concept of tissue engineering trachea (TET) was put forward to solve the problem. To better solve the problem of long-term airway stenosis caused by congenital airway stenosis, airway tumour, heart malformation, and other diseases, the concept of the tissue-engineered trachea (TET) was put forward as a new idea [6]. Seeding cell, tracheal scaffold, and effective blood supply are the three elements of TET, among which seeding cell is the core element and scaffold material is the basis. In this study, bone marrow mesenchymal stem cells were chosen as seeding cells. With their multidirectional differentiation potential, we have successfully differentiated them into airway epithelial cells [7] and chondrocytes [8], respectively. In addition, the acellular trachea is well prepared as the scaffold material [9] for this study (Figure 1).

In 2008, Zhang et al. in the UK reported a case of airway transplantation in 12-year-old children using tissue-engineered tracheas [10], which has obtained good results in a follow-up of up to five years. The application of the tissue-engineered trachea in children was realized. However, postoperative stenosis occurred during the follow-up. Respiratory distress caused by narrowness, granuloma formation, etc. should be treated by repeated hospitalization, interventional dilation, and stenting, thereby resulting in high cost and other problems [11, 12]. Local necrosis or infection of the trachea after transplantation is the cause of these phenomena. The main reason for this is the lack of ‘effective blood circulation' [13], which leads to the failure to repair damaged tissue in good time.

In the research of tissue engineering vascularization, the researchers proposed technologies such as cell implantation [14], suspension drop technology [15, 16], cell membrane technology [17, 18], myocutaneous flap technology [19, 20], and arteriovenous loop technology [21] to promote the regeneration of the blood vessels *in vivo*. Professor Okano's team in Japan [22–24] has prepared ultrathin poly(N-isopropyraclamide) materials. The cell membrane can be obtained directly by separating the cell from the culture dish through temperature changes, damaging the intercellular connections. Thus, the three-dimensional structure obtained in the original state of the cells was preserved, thereby promoting graft. This cell membrane technology has been reported in tissue engineering for the heart, liver, cornea, kidney, tendon, skin, and bone [25–31]. The membrane was cultured with cardiomyocytes, fibroblasts, and myoblasts to form a vascular network that could connect with the host. Cerqueira et al. [32] implanted the membrane prepared by keratinocytes and skin microvascular endothelial cells into a whole layer in mice with skin injuries. The results showed that the membrane group had a fast epithelial coverage and the local vascular density increased significantly. Though the membrane technology is of great importance to the equipment and materials, it is difficult to achieve popularization.

Much more than just an antioxidant, ascorbic acid (AS) is an essential molecule which works as a cofactor for many enzymes, such as prolyl hydroxylase (PH) and lysyl hydroxylase (LH), which are indispensable for collagen biosynthesis, and can enhance the activities of various hydroxylases and oxidases [33]. Also as extensively reported in the literature, AS is a molecule involved in the extracellular matrix secretion and in the osteogenic induction by modifying collagen molecules in posttranslational to make up components of the ECM of mesenchyme-derived tissues [34]. To approve that, a research aimed to evaluate the role of AS supplementation in a novel 3D-scaffold in vitro in which bovine pericardium collagen membrane BioRipar (BioR) was functionalized with human gingival mesenchymal stem cells (hGMSCs) that was treated with different concentrations of AS [35]. Besides, as for the inflammatory proliferation problem that occurred in the translation of TET, whether artificially synthesized scaffold is used, researchers confirmed that AS could reduce the detrimental result promoted by methacrylate in clinical dentistry, reduce the proinflammatory cytokine, and downregulate reactive oxygen species (ROS) production and NFkB/pERK/ERK signaling path [36]. Based on studies mentioned above, we believed that AS possessed the ability to make the induction from mesenchymal stem cells to stem cell sheet.

Therefore, in this study, we use ascorbic acid to induce bone marrow mesenchymal stem cells and construct stem cells *in vitro*. The structural characteristics of the diaphragm are discussed. In addition, we tried to build prevascularization *in vitro* by adding vascular factors to form a stem cell membrane, in combination with the new enzyme detergent cell removal method previously developed by our research team, which is made up of composite tissue engineering scaffolds by wrapping the trachea with a stem cell membrane, simulating all levels of the trachea. The middle microvascular distribution is aimed at promoting its vascularization. The potential of vascularization was explored through *in vivo* transplantation, so as to promote the research of tissue-engineered trachea vascularization and the research of tissue-engineered tracheas in the treatment of infant airway stenosis.

## 2. Materials and Methods

### 2.1. Isolation and Culture of Bone Marrow Mesenchymal Stem Cells (BMMSCs)

All animal experiments in our study were sponsored by the Animal Committee of Guangdong Provincial People's Hospital and carried out in accordance with its guidelines. Rabbit bone marrow mesenchymal stem cells (BMMSCs) were obtained according to Delorme et al.'s report [37, 38] with its characteristic of adhering to the plastic of the cell culture plate. After weighing the New Zealand rabbit, we first exposed and sterilized the ear vein, and then slowly injected 2 ml/kg of 1% pentobarbital solution (ZhiXing, China) through the ear vein. Secondly, we sterilized the skin of the sacroiliac joint to the femur of the rabbit and punctured the long bone with a 30 ml or 50 ml syringe needle, aspirating the femur bone marrow of the rabbit for about 2 ml. We mixed the bone marrow liquid suction with an *α*-MEM (Gibco, USA) and filtered impurities with a 200-micron nylon mesh (BD, USA). The BMMSC sediment was obtained after centrifugation (1500 r/min). Cells were seeded in culture flasks in an OriCell™ complete rabbit bone marrow mesenchymal stem cell culture medium (Cyagen, USA). Nonadherent cells were removed by medium change 48 h later. The adherent cells were kept in standard culture conditions (5% CO_2_ in humid air, 37°C), with the medium changed every three days. The cells were passaged using 0.25% Trypsin-EDTA (Gibico, USA) until approximately 90% confluence was reached. In our study, flow cytometry confirmed that the expression of CD44 (AbD, USA) in BMMSCs was over 90%, while that of CD31 (Novusbio, USA) or CD45 (AbD, USA) was negative [37]. BMMSCs of passage 3 were selected for experiments.

### 2.2. Fabrication and Characterization of the SCS Induced by Ascorbic Acid

The BMMSCs were first cultured in a cell culture dish at a cell density of 1 × 10^6^/cm^2^ in an OriCell™ medium. When certain cell confluence was reached, the medium was changed to an *α*-MEM with 20 *μ*g/ml ascorbic acid (Sigma, USA) [39] and 10% foetal bovine serum (Gibco, USA). The medium was changed every three days to promote the production of an extracellular matrix to form the cell sheet. After 14 days of culture, the cells at the edge of dishes would wrap, which implied that the stem cell sheets had formed and could be detached for further experiment. To investigate the general structure, the stem cell sheet was made into a paraffin slide and stained with hematoxylin-eosin and Masson [8].

### 2.3. Immunohistochemistry of the SCS

In order to figure out the angiogenic potential of the stem cell sheet, immunohistochemistry staining was performed. The samples were deparaffinized, rehydrated, and then immersed in 3% H_2_O_2_ for 20 min at RT to remove the intrinsic peroxidase activity. After washing, the samples were blocked using a goat serum working solution (Bioss, C-0005) for 15 min at RT. The samples were then incubated with mouse anti-rabbit CD31 antibody (1 : 100 dilution) overnight at 4°C. The samples were then stained using DAB (DAKO, K5007) according to the manufacturer's instructions. Finally, the cell nuclei were stained with hematoxylin for 1 min and then extensively washed with PBS. BMMSCs seeded on the well plate were used as a control. A positive reaction resulted in brown staining.

### 2.4. Real-Time RT-PCR of the SCS

For *in vitro* evaluation of gene expression related to vascularization, total RNA was isolated using TRIzol reagent (TaKaRa RNAiso Plus, 9108). Then, the total RNA was reverse-transcribed with a reverse transcription kit (TaKaRa, RR037A). Real-time PCR was performed with a PCR instrument (Bio-Rad, C1000) using SYBR Green PCR Master Mix (TaKaRa, RR420A). The PCR parameters were set as follows: 95°C for 15 s, followed by 60°C for 60 s. The gene primers used in this study are listed in Table 1; GADPH primers were used to normalize samples. The results were evaluated with the IBM SPSS Statistics 19.0 software program. All experiments were conducted in triplicate (Table 1).

### 2.5. Fabrication and Characterization of a Decellularized Tracheal Scaffold

The decellularized tracheal scaffold was digested with the Trypsin-EDTA (Gibico, USA) together with the detergent-enzymatic method [9] we reported previously. Shortly, the trachea was obtained, and the surrounding fascia was removed. Then, the trachea was digested in 0.25% Trypsin-EDTA (Gibico, USA), digestion B composed of Tris base, digestion C composed of Tris base and triton-A, and Digestion D composed of DNAse (Roche, Switzerland) and RNAse (Amresco, USA), in order. Finally, the scaffold was dehydrated in 75% ethanol. The decellularized trachea scaffold was prepared into paraffin sections, which were stained with HE to evaluate the efficiency of decellularization.

### 2.6. Implant Stem Cell Sheet and Decellularized Tracheal Scaffold

In order to determine the vascularization of the stem cell sheet and decellularized tracheal scaffold *in vivo*, a subcutaneous implant was performed. Firstly, the decellularized tracheal scaffolds were cut into 24 squares with a length of 0.5 cm. Half of the squares were encapsulated by a stem cell sheet; these were considered the tissue engineering tracheal group (TET). The others were the decellularized tracheal group (DT). The stem cell sheets and decellularized scaffolds used were freshly produced. After the encapsulation, both the TET and DT groups were treated with an *α*-MEM with ascorbic acid (20 *μ*g/ml) overnight, to keep the cells alive, and implanted within 24 h.

On the day of the implant, the TET and DT groups were divided into 2-week groups and 4-week groups. Taken together, there were four groups: TET-2-week, TET-4-week, DT-2-week, and DT-4-week. For those in the 2-week groups, the scaffold was removed at two weeks after implantation, while it was taken out four weeks after implantation in the 4-week groups. To evaluate the vascularization potential of the scaffolds, heterotopic transplantation was adopted. The rabbits (*n* = 6) were anaesthetized, and their backs were shaved and sterilized to make four incisions so that we could implant four groups in the same rabbit to maintain the same environment. After the implant, we closed the incisions with silk thread.

### 2.7. Vascularization Evaluation with HE and Immunohistochemical Staining

At two and four weeks after implant, the rabbits were euthanized and the incisions were reopened to retrieve the scaffolds. Fixed in 4% polyoxymethylene (Servicebio, China) for 24 h, the scaffolds were embedded in paraffin and sliced into 5 *μ*m thick sections. To evaluate the presence of luminal structures containing red blood cells, conventional HE staining was carried out. For immunohistochemistry, sections were deparaffinized and digested by an antigen retrieval solution, and then, the sections were blocked by a 5% blocking serum (Servicebio, China) for 30 min at room temperature. Mouse 1 : 100 anti-rabbit VWF antibodies (Abcam, UK) were used. Biotinylated goat 1 : 500 anti-mouse secondary antibodies (Agilent, DAKO K5007, USA) and DAB substrate kit (Agilent, USA) were used in accordance with the instructions. The microvascular density (MVD) [40, 41] was counted to evaluate vascularization after implantation *in vivo*. In short, we observed eight random view fields (under 40x magnification) of the stained sections obtained from six individual rabbits (two fields per rabbit). Luminal structures containing erythrocytes were defined as microvessels in HE staining. In immunohistochemical staining, any single endothelial cell or cell clusters stained by antibodies, whether forming a lumen or not, that had a clear boundary with the surrounding tissues were counted as a microvessel. The MVD was reported as the average number of microvessels in each section and expressed as mean values ± the standard deviation.

### 2.8. Statistical Analysis

All the *in vitro* experimental groups were carried out in triplicate. For the *in vivo* studies, there were six samples in each group. Student's *t*-test analysis was adopted to analyze the differences between groups for the *in vitro* research. Paired *t*-test analysis was used to analyze the differences between groups for the *in vivo* research. A *p* value of less than 0.05 was considered a statistically significant difference.

## 3. Results

### 3.1. Cell Morphology of BMMSCs and the Stem Cell Sheet (SCS)

**Figure 2:**
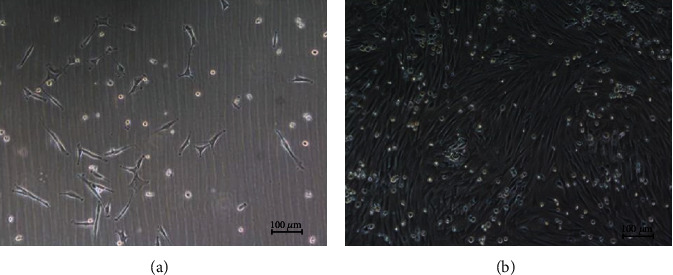
Morphology of rabbit bone marrow mesenchymal stem cells (100x). (a) After 3 days of culture, the adherent cells were in spindle and irregularly shaped, scattered around the bottle wall. A small number of nonadherent circular cells were found floating in the medium. (b) At days 8-10, the cell colony was formed and the cell density reached 90%.

**Figure 3:**
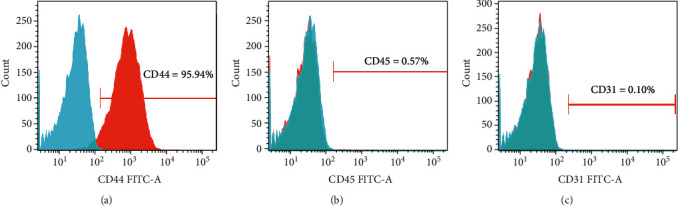
Flow cytometry in P1 bone marrow mesenchymal stem cells. (a) More than 95% of BMMSCs P1 were CD44 positive. (b, c) Less than 1% of BMMSCs P1 were CD 45 or CD 31 positive.

After three days of culture, the adherent cells were in a spindle and irregularly shaped, scattered around the bottle wall. There were also a small number of nonadherent circular cells floating in the medium. At days 8–10, the cell colony was formed, and the cell density reached 90%. After cell passage, a typical ‘fish group' colony was found at P1 (Figure 2). Flow cytometry demonstrated that more than 95% of these cells were CD44 positive, while less than 1% of them were CD45 or CD31 positive (Figure 3).

**Figure 4:**
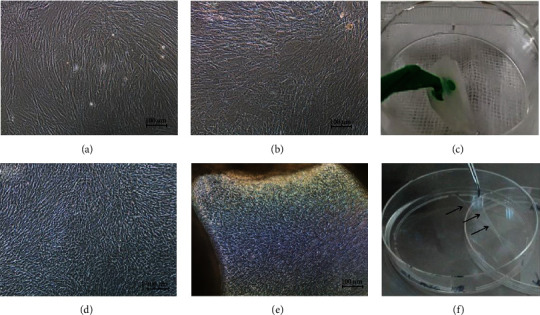
Morphology of BMMSCs P3 under intervention of ascorbic acid (100x). (a, b) BMMSCs P3 at day 5 and 14. (d, e) BMMSCs P3 under intervention of ascorbic acid at day 5 and 14. (c, f) The general structure of BMMSCs P3 without and with ascorbic acid (↙) at day 5 and 14.

Under intervention of ascorbic acid, the BMMSCs showed great proliferative ability. The cell density increased so fast that it was hard to distinguish the cell gap. For the control group without intervention of ascorbic acid, the shape of the BMMSCs became large and oval. Also, aging cells were found. At days 10–14 after the intervention of ascorbic acid, a hyaline membrane was formed and could easily be lifted up by a forceps. On the other hand, no membrane was visibly formed or even found by scraping in the control group (Figure 4).

**Figure 5:**
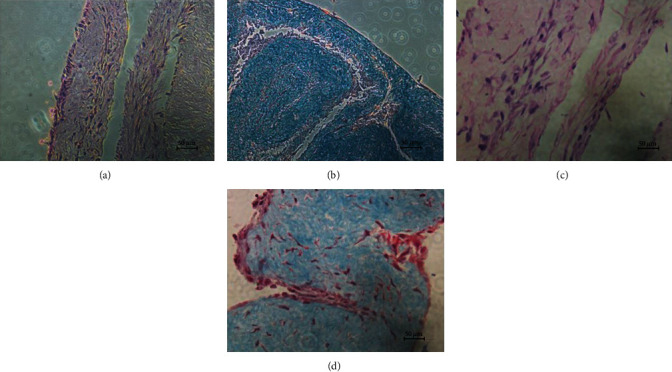
Morphology of BMMSC sheet under HE and Masson stain. (a, c) Stem cell sheet in HE staining under magnification of 100x and 400x. (b, d) Stem cell sheet in Masson staining under magnification of 100x and 400x.

For the cell sheet formed under ascorbic acid, there were a large number of cells whose cytoplasm was dyed pink and nucleus was dyed blue in HE staining (Figures 5(a) and 5(c)). In Masson staining, the cell sheet was composed of a large amount of extracellular matrix dyed blue (Figures 5(b) and 5(d)).

**Figure 6:**
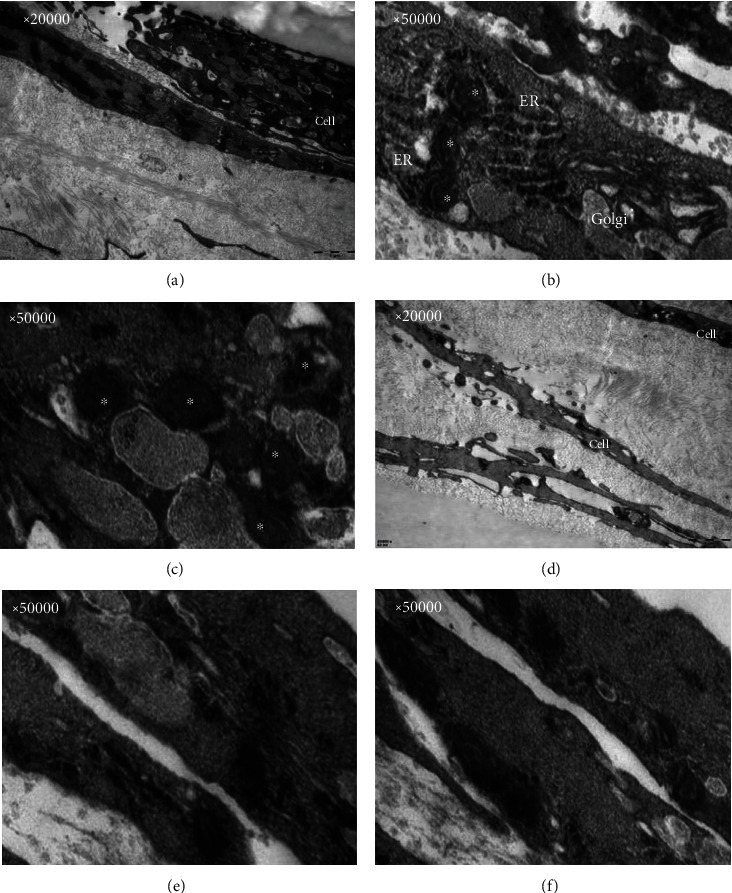
Morphology of BMMSCs and SCS in transmission electron microscope. A large amount of collagen structure was observed by transmission electron microscopy, together with numerous endoplasmic reticulum (ER), Golgi, and vesicle structure (^∗^) inside cells.

Under transmission electron microscopy, close intercellular connections were observed. Compared with the ultrastructure of the SCS and BMMSCs, the experimental group had abundant organelles and abundant mitochondrial structures, indicating active cell proliferation. In addition, a large number of rough endoplasmic reticulum and Golgi apparatus and dense vesicle-like structures were observed, which suggest enhanced secretion of BMMSCs; in contrast, the control group showed sparse organelles (Figure 6).

### 3.2. Vascularization Evaluation of the SCS

**Figure 7:**
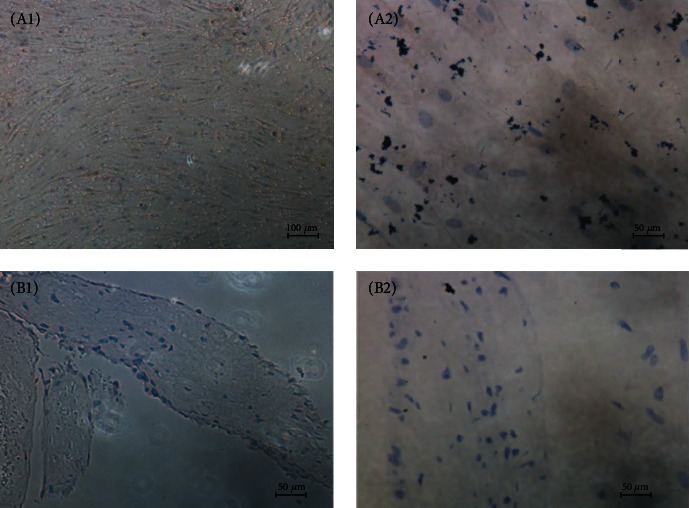
Immunohistochemical evaluation of CD 31 in BMMSCs and SCS. (A1, A2) Immunohistochemistry staining of CD 31 in BMMSCs under magnification of 100x and 400x. (B1, B2) Immunohistochemistry staining of CD 31 in SCS under magnification of 100x and 400x.

To assess the vessel formation ability of the SCS *in vitro*, immunohistochemistry staining was carried out using a monoclonal mouse anti-rabbit CD31. The results indicate that no vessel was formed in the SCS (Figure 7).

**Figure 8:**
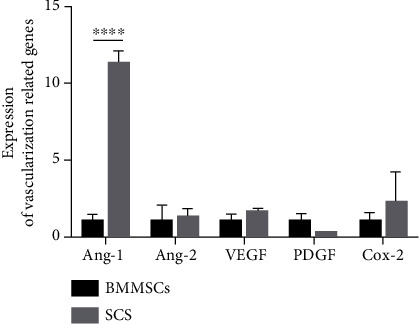
Expression of vascularization related genes in SCS and BMMSCs. ^∗∗∗∗^*p* < 0.0001. As compared with BMMSCs, higher expression of Ang-1 was observed in SCS, while no difference was noticed in other gens.

To value the gene expression that promotes vascularization, markedly increased expression of angiopoietin-1 (Ang-1), a growth factor that promotes angiogenesis, was observed in the SCS (*p* < 0.0001). For other pro- or antiangiogenesis gene expression, no difference was noticed between BMMSCs and the SCS (Figure 8).

### 3.3. Characterization of the Decellularized Tracheal Scaffold

**Figure 9:**
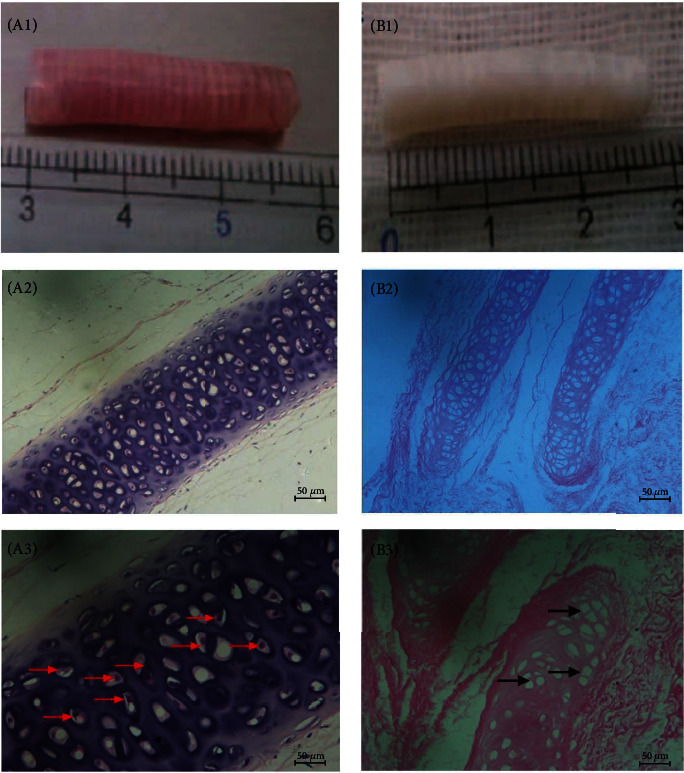
The general and histological morphology of tracheal before and after decellularization. (A1, B1) The color of trachea turned from pink to white after decellularization. (A2, A3) The nucleus of trachea before decellularization was in dark blue (red arrow) under magnification of 100x and 400x. (B2, B3) The nucleus was removed (black arrow) and only pink cytoskeleton left after decellularization under magnification of 100x and 400x.

After the decellularization of the trachea, the color of the trachea changed from pink to white, while there was no significant change in size or general structure. In order to evaluate the acellular efficiency, hematoxylin-eosin staining was carried out. HE staining confirmed that the blue nucleus was removed completely, with only a pink cytoskeleton and extracellular matrix left (Figure 9).

### 3.4. Postoperative Evaluation

During the four weeks of the experiment, all animals survived. There was no animal death because of operative adverse reactions caused by anaesthesia, operation, rejection, or infection. All six rabbits showed great increase in weight gain. After the subcutaneous transplantation, all the incisions healed well without any infectious signs such as swelling, suppuration, exudation, or rejection. The scar is barely seen at day 14 after surgery.

**Figure 10:**
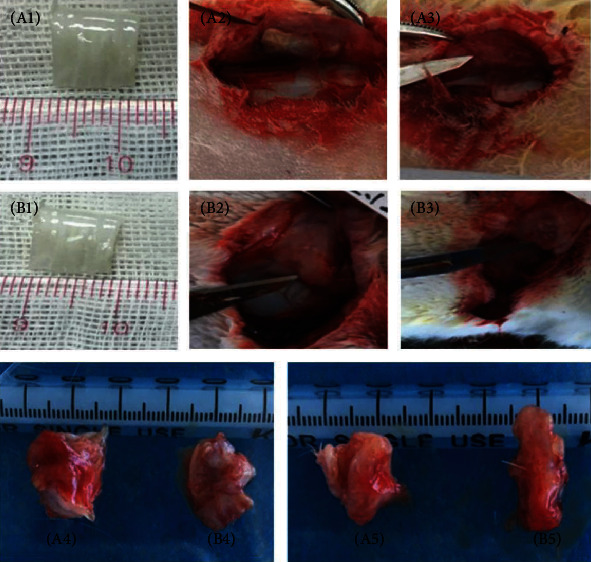
Morphology of tracheal scaffolds after transplantation. (A1–A5) DT's overall structure at day 0, 14, and 28 after subcutaneous transplantation. (B1–B5) TET's overall structure at day 0, 14, and 28 after subcutaneous transplantation. No significant difference was observed between both groups at the same time.

**Figure 11:**
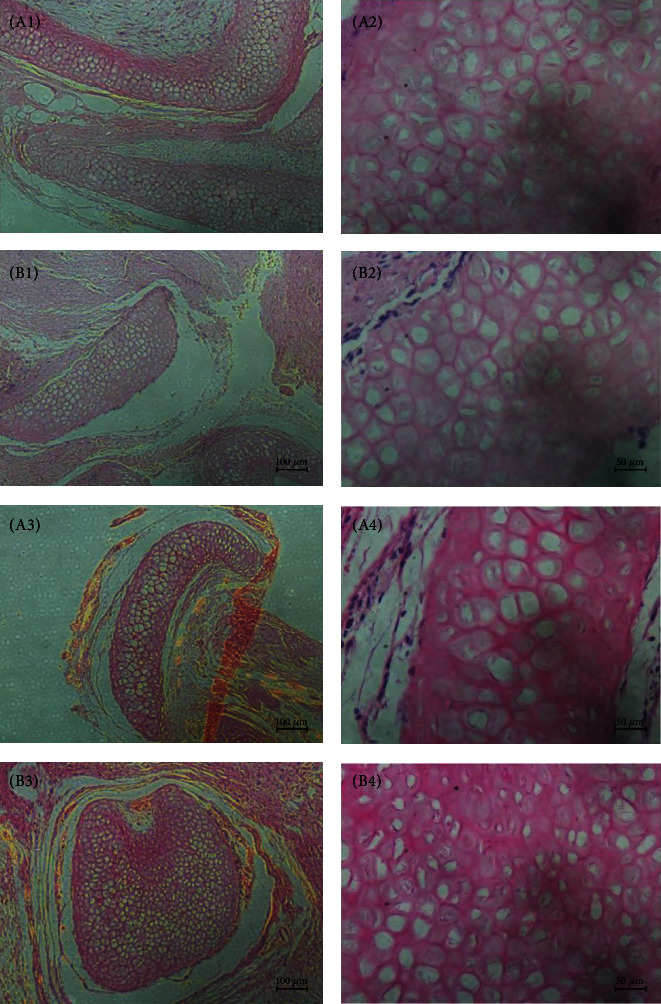
Histologic morphology of tracheal scaffold after transplantation in HE staining. (A1–A4) DT's microstructure at 2 and 4 weeks under magnification of 100x and 400x. (B1–B4) TET's microstructure at 2 and 4 weeks under magnification of 100x and 400x. The tracheal scaffold had kept its structure after transplantation.

Two weeks and four weeks after surgery, the incisions were opened again and the implants were taken out. The implanted scaffolds adhered to the surrounding tissues; thus, the boundary was blurred in both groups. The scaffolds shrank after transplantation, and the shape changed from square to irregular (Figure 10). HE staining showed that the tracheal scaffold had kept its structure after transplantation (Figure 11).

### 3.5. Vascularization Evaluation of DT and TET

**Figure 12:**
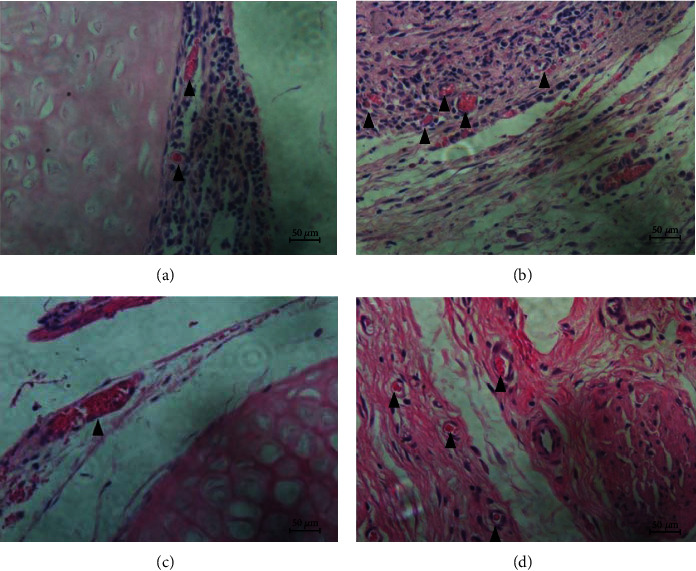
Microvessels around implanted trachea in HE staining. For HE staining, microvessels containing red cells can be seen (◤), and for different times, the TET groups had a significant upregulation of MVD than the DT groups; Within the same group, the discrepancy of results of between two weeks and four weeks was not statistically significant in statistics.

**Figure 13:**
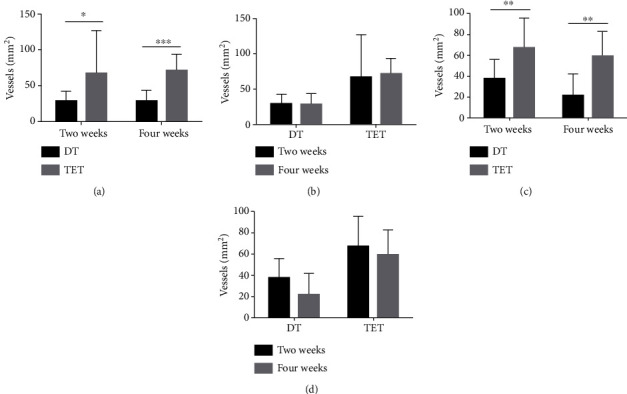
HE staining of microvascular density and immunohistochemical staining of vWF. (a) In each transplantation group at different time points. After two weeks and four weeks, TET groups had significant upregulation of MVD than DT groups (^∗^*p* < 0.05, ^∗∗∗^*p* < 0.0001). (b) Microvascular density at different time points in each transplantation group. In the same group, no significant difference was seen in different time points. (c) Microvascular density in each transplantation group at different time points. For the immunohistochemical staining vascular endothelial cell marker vWF secreted in different time points, the MVD in the TET groups was significantly increased than that in the DT groups (^∗∗^*p* < 0.001). (d) Microvascular density at different time points in each transplantation group. Within the same groups, for different times of two weeks and four weeks, there was no significant statistical difference in MVD counting.

HE staining was carried out for the evaluation of the MVD. At different time points, the TET groups had significant upregulation of MVD compared to the DT groups (for two weeks, *p* = 0.014; for four weeks, *p* < 0.0001). Within the same group, the discrepancy of results between two weeks and four weeks was not statistically significant (DT: *p* = 0.902; TET: *p* = 0.726) (Figures 12 and 13).

**Figure 14:**
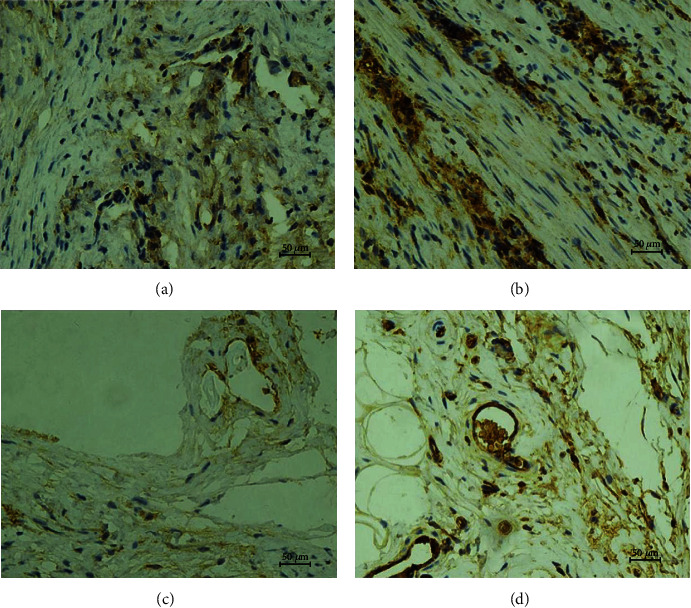
Immunohistochemical staining of vWF: microvessels around implanted trachea. For the immunohistochemical staining of different times, the MVD in the TET groups was significantly increased than that in the DT groups. Within the same groups, for different times of two weeks and four weeks, there was no significant statistical difference in MVD counting.

Immunohistochemical staining of von Willebrand factor (VWF) further confirmed the above results (for two weeks, *p* = 0.001; for four weeks, *p* = 0.001). Within the same groups, for the different time periods of two weeks and four weeks, there was no significant statistical difference in MVD counting (DT: *p* = 0.062; TET: *p* = 0.346) (Figures 13 and 14).

## 4. Discussion

Ascorbic acid (AA) is an antioxidant which has the abilities of enhancing cellular survival, promoting proliferation, preserving cellular immunophenotype, and differentiation. In Wei et al.'s experiment [39], AA was added into different sources of human bone marrow mesenchymal stem cells with a concentration gradient, which then significantly promoted cellular proliferation. In our study, rabbit bone marrow mesenchymal stem cells showed increased proliferation activity under the effect of low mass concentration (20 *μ*g/ml) ascorbic acid, as compared with the control group. AA promoted cell proliferation by increasing the telomerase activity of bone marrow mesenchymal stem cells. Also, the number of mitochondria and other functional organelles is increased, as seen under a transmission electron microscope. During subsequent culture, these cells grew closer and appeared as a ‘climbing layer.' The phenomenon of the ‘climbing layer' could be seen in histology as ‘layer upon layer.' However, bone marrow mesenchymal stem cells were a monolayer patch. In the process of cell proliferation, when the lamellar pseudopodia between cells make contact [42], the forward movement of the cells will gradually weaken due to the activation of the gene signaling pathway [43], and the movement direction will change as well. This phenomenon is called ‘contact inhibition.' Under the action of AA in our experiment, the phenomenon of cell contact inhibition was obviously reduced, and a phenomenon similar to ‘cell transformation' appeared.

When the cells were cultured for 10–14 days, a thin semitransparent membrane-like structure could be formed generally, which could be completely stripped without the action of digestive enzymes. Also, a large amount of collagen extracellular matrix components could be seen histologically. Under a transmission electron microscope, a rough endoplasmic reticulum and dense vesicle structure were also observed as the structural basis for cells to synthesize a large amount of extracellular matrix. In the 1980s, it was reported that AA has the function of promoting the synthesis of collagen polypeptide by skin fibroblasts [44]. Later, mounting evidence showed that AA has the same effect in chondrocytes, mesenchymal stem cells, and other types of cells. Wu et al. [45] proved that AA could also induce extracellular matrix reconstruction by promoting matrixmetallo proteinase-2 (MMP-2) activity and deposition of various types of collagen, thus maintaining the three-dimensional microenvironment around cells [46], which is in line with the three-dimensional lamellar structure formed in our study.

Serial analysis of gene expression and metabolome analysis suggested that the downstream genes of hypoxia-inducible factor 1-A (HIF1*Α*) are unregulated and there is a conversion to hypoxia-mimetic metabolism due to HIF1*Α* accumulation, which is caused by decreased enzyme activity in an AA-free medium. AA promotes HIF1*Α* breakdown and activates mitochondria, affecting cell proliferation and metabolism. Comprehensive evaluation of the effects of AA on various metabolic products in MSCs revealed that AA increases HIF1*Α* hydroxylase activity, thereby suppressing HIF1*Α* transcription and resulting in mitochondrial activation [47].

With the administration of AA, the gene expressions of fibronectin and type I collagen have changed. Kim et al. [48] demonstrated the decrease of Collagen Type I Alpha 1 deposition, the increase of Collagen Type V Alpha 1 and Collagen Type VI Alpha 1 proteins in fat precursor cells with AA, indicating that ascorbic acid affects the differentiation of fat precursor cells by modifying the expression of a different extracellular matrix. The extracellular matrix includes fibronectin, collagen types I–IV, and laminin, which are synthesized and secreted by cells themselves. It not only provides structural support for cells and tissues but also serves as a necessary microenvironment for cellular growth, development, and differentiation. We used to believe that biochemical factors affected the differentiation of cells. However, we now find that biophysical signals are also regulated.

Shona Pek et al. [49] extended their experiments to the three-dimensional culture mode and came to the very same conclusion. In the field of three-dimensional cultivation, Wingate et al. [50] also found that bone marrow mesenchymal stem cells have a tendency to differentiate into vascular endothelial cells and vascular smooth muscle cells. The extracellular matrix plays an important role in the process of the cell's perception of mechanical stimulation and information transmission through its different hardness. It influences the differential pathway through the Rho family protein, protein kinase C, integrin, and other signal pathways and determines its ‘life journey' [51].

In the past, various synthetic collagen components, such as gelatin and polyacrylamide hydrogel, were added during cell culture to study the effect of the extracellular matrix on cell differentiation [52], while few have focused on the effect of an extracellular matrix synthesized by cells themselves on cell differentiation. Studies have shown that basic fibroblast growth factor, insulin-like growth factor 1, and other cytokines can promote the expression of extracellular matrix Palmitoyl Pentapeptide-3, which could facilitate the production of extracellular matrices such as collagen and fibronectin [53]. However, these molecules are mostly cytokines or biological materials, which are difficult to obtain and expensive.

After AA intervention, rabbit bone mesenchymal stem cells form a three-dimensional lamellar structure from scattered cells. In tissue engineering, the selection of scaffold materials had always been an important problem perplexing scientists, as it requires a certain biocompatibility and plasticity of materials [54]; the ‘membrane' formed in this experiment was undoubtedly a natural scaffold material for bone marrow mesenchymal stem cells and completely preserves the microenvironment between cells. In a practical application, a cell membrane has been reported in the repair of a corneal epithelium, the repair of the mucosal surface epithelium of internal organs and organs of the cavity, and the reconstruction of the vascularization of ischemic and necrotic tissues [18, 55]. Moreover, compared with the cell membrane prepared by special physical and chemical materials in the past, using AA alone greatly reduces the cost and technical difficulty.

Above all, under the action of AA, the proliferation ability of rabbit bone marrow mesenchymal stem cells is significantly enhanced, and the transformation effect resisting the phenomenon of ‘contact inhibition' appears. Meanwhile, it promotes the secretion and synthesis of the extracellular matrix. These cells could form a ‘membrane' structure, which is conducive to the complete preservation and acquisition of the whole cellular and extracellular matrix structure and has great significance in the research of tissue engineering. AA has a certain specificity for the synthesis of different extracellular matrix components, and its application in cell differentiation and tissue engineering still awaits further exploration.

In the study of tissue-engineered vascularization, there are two vascular construction strategies: *in vivo* and *in vitro* [56]. Most studies [31, 57–59] mainly focus on the joint between endothelial cell and bone marrow mesenchymal stem cell coculture to induce the formation of blood vessels, while few have employed the administration of an exogenous vascular factor. Therefore, in our study, starting from the construction of simple mesenchymal stem cell membranes, we tried to use them as scaffolds to provide a vascularized microenvironment and to promote vascular regeneration through vascularized induction.

In this study, the BMMSCs and SCS were induced with endothelial growth medium-2 (EGM-2). Notably, the expression of CD31, a marker of angiogenesis, was found to be negative, indicating that the expression of CD31 in cells and tissues was not successfully induced by EMG-2. Also, the CD31 expression was negative when an endothelial cell growth supplement (ECGS) is added to the culture of the vascularized SCS. Liang et al. [60] showed that the differentiation of BMMSCs into vascular endothelial cells requires the induction of the surrounding collagen matrix and other three-dimensional microenvironments. Moreover, temporal differences were noticed regarding the expression of CD31, CD34, Flk1, and other cell markers. It can be seen that cellular microenvironment, biological factors, relative dosage and action time, etc. should be considered in the construction of the tissue diaphragm, so that it can successfully express vascularization marker proteins. That still needs further research and exploration.

In the expression of vascularized genes, EMG-2 induction showed that the expression of vascular endothelial growth factor (VEGF) in the SCS group was significantly higher than that in the BMMSC group. As a signaling protein that stimulates angiogenesis and increases capillary intensity in the vascular network, VEGF [61] plays an important role in vascular growth and angiogenesis. *In vitro* studies have shown [62] that VEGF can effectively stimulate angiogenesis by facilitating the proliferation and migration of endothelial cells, eventually forming a tubular network similar to capillaries. Undoubtedly, VEGF is involved in various signaling cascades in endothelial cells. When binding to vascular endothelial growth factor receptor 2, VEGF initiates a cascade of tyrosine kinase signaling reactions, thus stimulating changes in vascular permeability, proliferation/survival migration, and eventual differentiation into mature vessels.

AA significantly promoted the expression of angiotensin-1 (Ang-1) [63], a factor promoting angiogenesis, in the SCS. When the ECGS factor was added, the angiotensin-2 (Ang-2) gene expression was also increased. Ang-2 [64] is an antagonist of the Ang-1-Tie system. Therefore, it seemed that ECGS had an effect on both promoting and inhibiting vascularization. In the process of angiogenesis, Ang-1 has a linkage effect with VEGF, promoting vascular remodeling and maturation, preventing vascular leakage [65], and maintaining its normal physiological functions. Hammes et al. [66] found that Ang-2 can change the permeability of blood vessels and facilitate the infiltration and migration of protease, cytokines, and angiogenesis precursor cells, thus promoting the initiation of angiogenesis in blood vessels. Therefore, we believe that Ang-1 and Ang-2 could promote vascularization, which contributes to the improvement or initiation of vascular tissue engineering.

Above all, though the induction of both EGM-2 and ECGS failed to upregulate the CD31 expression, the gene expressions of VEGF, PDGF, COX-2, and Ang-1 had increased, indicating its potential application value in tissue engineering vascularization. However, its specific mechanism still awaits further exploration.

Vascular endothelial cells or a variety of cells with multidirectional differentiation potential were selected as the original materials for local microvascular construction. In this study, allogeneic bone marrow mesenchymal stem cells (BMMSCs), well known with their multidirectional differentiation potential, were selected as seeding cells [67].

In terms of assembly technology, three-dimensional scaffolding materials [68], cell sheet technology [69], nanocoating technology [70], and 3D bioprinting technology [71] are currently four commonly used strategies. Gao et al. reported that a tissue-engineered trachea, based on a 3D-printed poly(L-lactic acid) scaffold which has a similar morphology to the native trachea of rabbits, was used for long-segmental tracheal reconstruction [72]. However, when the slow degradation rate of the 3D-printed poly(L-lactic acid) scaffolds is considered, the therapeutic effect remains unclear in infants as their tracheas grow with age. In the study of Zhang et al. [73], a double-cell sheet (DCS) complex, composed of an osteogenic cell sheet and a vascular endothelial cell sheet, developed an osteogenesis and blood vessel formation potential in BALB/c nude mice, which are immunodeficient. There are two main problems: the cost is high, and the immunogenicity of the DCS complex is uncertain. In our study, the combination of cell sheets and decellularized tracheal materials was used to promote vascular regeneration. The three-dimensional structure of stem cell sheets could be obtained under the action of ascorbic acid, without enzymatic digestion. Also, it completely retains the connection between cells, the extracellular matrix composed of various collagens, and other components. In the study of bone marrow mesenchymal stem cells, the cell fate is determined not only by the surrounding biochemical factors but also by the microenvironment [74] formed by the vesicles synthesized and secreted by cells themselves as well as the physical environment [75].

After transplantation, the stent was ‘perfused' *in vivo*. Compared with the group of tracheal stents alone, HE and immunohistochemical VWF staining both confirmed the significantly increased microvascular intensity of the graft after implantation of the composite diaphragm tracheal stent *in vivo*, indicating that the acellular tracheal scaffold combined with the stem cell sheet promoted its angiogenesis ability. On the other hand, no obvious difference was seen in the comparison of time points, which may be due to the selection of experimental nodes and the time points of local vascular regeneration and microangiogenesis. Villar et al. [76] confirmed that the living cell membrane is composed of a large number of collagens. Also, the cells have the potential to promote the migration, proliferation, and secretion of EGF, IL-6, Ang, and other vascular regeneration-related factors. It also induces increased production and secretion of most of the angiogenic factors, including EGF, IL-6, and Ang. In addition, human umbilical vein endothelial cells were added, and the subsequent regeneration of the blood vessels was confirmed. Sasagawa et al. [77] considered that the construction of a cell sheet containing vascular endothelial cells not only realizes *in vitro* vascularization but also promotes rapid revascularization after transplantation.

In conclusion, the study of stem cell sheets to promote graft vascularization is of great significance, not only in promoting host vessel access to the graft but also in cultivating its internal microvasculature, which provides a promising direction for tissue engineering trachea vascularization.

## 5. Conclusion

A tissue-engineered trachea was generated from a cell sheet formed under the induction of ascorbic acid as well as a decellularized trachea. Through experiments *in vitro*, it was verified that the cell sheet can promote vascularization at the gene level. *In vivo* experiments confirmed that the composite tissue-engineered trachea has a stronger vascular regeneration potential. Secondly, the source, mechanism, speed, and specific morphology of vascular network formation have not been further clarified in this study. They still remain to be explored in the future.

## Figures and Tables

**Table 1:** List of primers used in real-time PCR.

Primer ID	Sequence (50–30)	Expect size (bp)
GAPDH-F	TGACGACATCAAGAAGGTGGTG	121 bp
GAPDH-R	GAAGGTGGAGGAGTGGGTGTC	
Ang-1-F	CAAATGTGCCCTCATGCTTAC	121 bp
Ang-1-R	GTGCCACTTTATCCCGTTCA	
Ang-2-F	GCGTTGATTTCCAGAGGACG	258 bp
Ang-2-R	GGCTGATGCTGCTTATTTTGC	
VEGF-F	GCAATGATGAAAGCCTGGAGTG	188 bp
VEGF-R	CTTGCCCTTTCCTCGAACTGAT	
PDGF-F	TGGACACCGTCAATGTCACC	191 bp
PDGF-R	ACAGTCTGTGGGTTTCTGCTT	
COX-2-F	TCTACCCGCCTCACATCCCT	162 bp
COX-2-R	GCTGCTCATCATCCCATTCTG	

## Data Availability

The datasets used and analyzed during the current study are available from the corresponding author on reasonable request.
